# Mechanical Performances of Al-Si-Mg Alloy with Dilute Sc and Sr Elements

**DOI:** 10.3390/ma13030665

**Published:** 2020-02-02

**Authors:** Zichen Zhang, Qingfeng Zhao, Lihua Liu, Xingchuan Xia, Cheng Zheng, Liwei Quan, Jian Ding, Xueguang Chen, Xudong Luo, Lisheng Wang, Kaihong Song, Chong Li, Yongchang Liu

**Affiliations:** 1 School of Material Science and Engineering, Hebei University of Technology, Tianjin 300401, China; zhangzc6666@163.com (Z.Z.); imzhaoqf@126.com (Q.Z.); zc1752829531@163.com (C.Z.); 13292509244@163.com (L.Q.); 2 CITIC Dicastal Co., LTD, Qinhuangdao 066011, China; liulihua@dicastal.com (L.L.); wanglisheng@dicastal.com (L.W.); 3 School of Material Science and Engineering, Tianjin University, Tianjin 300072, China; lichongme@tju.edu.cn; 4 Tianjin Zhongwang Aluminium Co., LTD, Tianjin 300300, China; 17596530308@163.com

**Keywords:** Al-Si-Mg alloy, precipitation behavior, heat treatment, mechanical performance

## Abstract

Due to its excellent comprehensive performances, Al-Si-Mg alloy i widely used in automotive, transportation and other fields. In this work, tensile performances and fracture behavior of Al-Si-Mg alloy modified by dilute Sc and Sr elements (Al-7.12Si-0.36Mg-0.2Sc-0.005Sr) were investigated at the temperature of −60–200 °C for the first time, aiming to obtain a satisfactory thermal stability within a certain temperature range. The results showed that the new designed Al-Si-Mg alloy possessed a completely stable yield strength and a higher-level elongation under the present conditions. Fracture morphology analysis, fracture profile observation and strengthening mechanism analysis were applied to elucidate the evolution mechanisms of yield strength and elongation of the alloy. The fracture modes were significantly distinct in different temperature sections, and the reasons were discussed. In addition, the interaction among the nano precipitate phase particles, the deformation substructure and the dislocations were responsible for the thermal stability of the alloy within a certain temperature range.

## 1. Introduction

In the past few decades, due to its lightweight, excellent mechanical performances, good corrosion resistance and castability, hypoeutectic Al-Si casting alloy, especially Al-Si-Mg alloys, were widely used in automotive engines, high-speed train sleepers and other transportation fields [1,2,3]. It is known that automotive engines operated at the temperature of up to 200 °C [4], and the service temperature of high-speed train sleeper materials may reduce to −60 °C in northern China [5]. However, Al-Si-Mg alloy will lose its strength above 150 °C [6] and generate low-stress fracture due to local plastic deformation caused by stress concentration at low temperatures [7], leading to the demand for expanding its operating temperature range from −60 °C to 200 °C [8]. 

It is known that mechanical performances of Al-Si-Mg alloy are greatly affected by temperature, which is not conducive to its application. Yield strength (YS or $\sigma_{s}$) is the most basic parameter of alloy in practical applications and part design [9]. Therefore, how to stabilize YS at different temperatures to improve service safety is crucial but challengeable for extended applications of Al-Si-Mg alloy. While, up to date limited investigations focused on the YS stability of Al-Si-Mg alloy in a wide temperature range. Giovanni et al. reported that the ultimate tensile strength (UTS) and YS decreased by 30.36% and 21.74%, respectively, for the T6 treated A356 alloy which was modified by Ni/V when the temperature increased from 20 to 235 °C [6]. Rahimian et al. revealed the role of Zr in Al-Si-Cu-Mg alloy utilized at elevated temperatures, the results showed that UTS and YS of T6 treated Al-Si-Cu-Mg-Zr alloy decreased by 34.04% and 31.18%, respectively [10]. Ma et al. investigated the variation of tensile performance of AlSr5 modified and T6 treated A356 alloy at −60 °C~20 °C, found that UTS and YS decreased by 7.80% and 6.80%, respectively [11]. 

It can be seen that modification of the morphology of eutectic Si and refine the grain size of a-Al cannot improve the YS stability of the alloys effectively. Previous research showed that due to the stability of Al_3_Sc phase at elevated temperatures, Sc is arguably known as one of the most attractive and effective microalloying elements to develop high-performance aluminum-based alloy for high temperature applications [2,12]. Karnesky et al. suggested that due to the strain field originating from lattice and modulus mismatch between Al_3_Sc and the matrix alloy, uniformly dispersed Al_3_Sc phase can strengthen the alloy, [13]. Gao et al. obtained the coarsening-resistant Al_3_Sc phase in diluted Sc microalloyed Al-Cu alloy through multistep aging treatment and found Al_3_Sc phase can improve the thermal stability of matrix alloy at 250 °C [14]. Tzeng et al. investigated the effect of dilute Sc and Be elements on the thermal stability of Al-7Si-0.6Mg alloy, and the results showed that precipitation of fine Al_3_Sc particles effectively inhibited grain growth and suppressed dislocation movement, leading to a better thermal stability at the thermal exposure temperature of 250 °C [15]. Qiu et al. investigated the effect of Sr and La elements composite modification on the mechanical performances of Al-Si-Mg alloy; the results indicated that Sr can promote the more isotropic growth of eutectic Si and convert the morphology of eutectic Si from acicular-like to short fibrous or fine particles, which promoted the mechanical performance of the alloy [1]. Xu et al. performed Sc and Sr elements composite modification and T6 treatment on Al-Si-Mg alloy, focused on the mechanical properties at room temperature and obtained satisfactory UTS (296 MPa) and El. (14.4%). The mechanism of modification and strengthening by Sc and Sr elements was clearly explained; the research has significant implications for the application of Sc and Sr elements [16].

Though Sc element can improve the high temperature performances of Al-Si-Mg alloy, few literatures focused on the effect of a wide range of temperature variations on the YS stability. Based on this, diluted Sc and Sr elements were used to modify Al-Si-Mg alloy (Al-7.12Si-0.36Mg-0.2Sc-0.005Sr, wt. %, hereinafter refers to wt. %) in this work, aiming to investigate its mechanical performances (particularly the YS) at a temperature range of −60 °C to 200 °C. The results indicated that the alloy possessed satisfactory YS stability over a wide temperature range, and the reasons were discussed through fracture behavior analysis, microstructure observation and strengthening mechanism analysis.

## 2. Experimental Procedures

### 2.1. Materials and Specimen Preparation

Pure aluminum (99.99%), Al-20Si, Al-10Mg, Al-2Sc and Al-10Sr master alloy were used as the matrix to prepare Al-Si-Mg-Sc-Sr alloy with the nominal composition of Al-7.12Si-0.36Mg-0.2Sc-0.005Sr. Detailed preparation processes were as follows: (1) melt a certain quantity of pure aluminum at 740 ± 1 °C within a graphite crucible in a resistance furnace. (2) degass the melt for 5 min with high purity argon gas and removing the slag immediately. (3) add a certain amount of Al-20Si, Al-10Mg, Al-2Sc and Al-10Sr master alloy into the melt and holding for 20 min, degass the melt for 5 min with high purity argon gas and removing the slag immediately. (4) pour the melt to a permanent mold (with inner diameter and height of 50 mm and 160 mm, respectively, and preheated to 200 °C) and cooling the ingot to room temperature in the air. After that, specimens were solution treated at 540 °C for 280 min in a resistance furnace with the temperature accuracy of ±1 °C and then immediately transformed to another resistance furnace at the temperature of 150 °C for 5 h.

### 2.2. Mechanical Performances Test

Specimens for tensile tests were prepared according to GB/T228.1-2010, GB/T228.2-2015 and GB/T13239-91 standards, respectively. Electronic Universal Material Testing Machine (UTM5105X, Xiamen Panson Electronic Technology Co., Ltd., Xiamen, China) was used for tensile tests at the temperature range of −60–200 °C. During the tests process, specimens were cooled or heated in an insulation tank for 10 min to obtain a uniform temperature throughout the specimen. Displacement control was used to execute the whole process with a crosshead speed of 1.5 mm/min. To ensure the reproducibility, at least four specimens were tested for each parameter and the average data were used.

### 2.3. Microstructure Observation

Specimens for fracture microstructure observation were taken from fractured specimens by an electro-discharging machine. Fracture of the specimen was wrapped in cotton and kept 10 mm distance from incision to ensure the integrity of fracture then ultrasonic cleaned with ethanol to remove any surface dirt or oils. The fracture profile specimens for metallographic observation were sectioned perpendicular to the fracture surface carefully and ground through successive grades of silicon carbide abrasive papers up to 2000 grit, polished using 2.5 μm diamond polishing paste, then ultrasonic cleaned with alcohol and dried with cold flowing air in the fuming cupboard. The polished specimens were first etched with 0.5 vol % HF for 30 s and then washed with distilled water and ethanol, at last dried with cold flowing air. The fracture microstructure was examined by a Hitachi S-4800 Field Emission Scanning Electron Microscopy (FESEM, Tokyo, Japan) with an accelerating voltage of 20 kV, and the microstructure of fracture profile was examined by a Lecia DM2700M Optical Microscope (OM, Wetzlar, Germany).

Specimens for Transmission Electron Microscope (TEM) observation were obtained at the tensile fracture distance of 0.5 mm and a slice with the thickness of 300 μm were cut by an electro-discharging machine. Then mechanically ground to about 50 μm, punched into 3 mm wafers and finally twin-jet electro polished to 50–150 nm with 30 vol % nitric acid methanol at −30 °C. Particle morphology and dislocation distribution were observed by a Tecnai F30 TEM (FEI, Hillsboro, Oregon, USA) with an accelerating voltage of 200 kV.

## 3. Results and Discussion

### 3.1. Mechanical Performances

**Figure 1:**
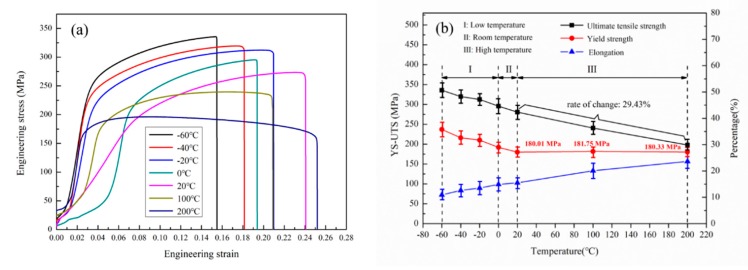
Engineering stress-strain curves of the alloy at different temperatures (**a**) and mechanical performance statistic results (**b**).

Typical engineering stress-strain curves of T6 treated Al-7.12Si-0.36Mg-0.2Sc-0.005Sr alloy at different temperatures are shown in Figure 1a, and specific values of mechanical performances are given in Figure 1b. As shown in Figure 1a, the tensile deformation curves are composed of elastic deformation portion, plastic deformation portion and fracture portion. For accurate description, test temperatures were divided into three sections: (I) low-temperature: −60–0 °C, (II) medium-temperature: 0–20 °C and (III) high-temperature: 20–200 °C, respectively. From Figure 1b, it could be seen that the UTS keeps decreasing with the temperature elevating from −60 °C to 200 °C, and the change proportion is about 29.43%. As for the YS, it shows a mild decreasing tendency in low-temperature and medium-temperature section; however, it appears to be a completely stable phenomenon in high-temperature section. The YS values at 20, 100 and 200 °C are about 180.01 MPa, 181.75 MPa and 180.33 MPa, respectively, indicating that this alloy possesses excellent yield strength stability at the temperature range of 20–200 °C. The elongation (El) increases from 10.9% to 23.63% with the temperature increasing from −60 °C to 200 °C. It is worth noting that the decreased amplitude of UTS is basically the same compared with the result of other researchers [6,10,11,16], and El stabilizes at a higher level, exhibiting that the alloy possesses satisfactory plasticity as well.

### 3.2. Fracture Morphology Analysis

**Figure 2:**
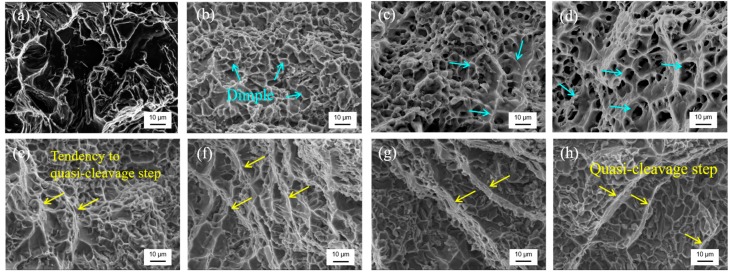
Fracture morphologies of the untreated alloy (**a**) [1] and Sc-Sr composite modified alloy under different temperatures: (**b**) 20 °C, (**c**) 100 °C, (**d**) 200 °C, (**e**) 0 °C, (**f**) −20 °C, (**g**) −40 °C, (**h**) −60 °C.

Representative fracture morphologies of the samples tested at different temperatures are shown in Figure 2. Compared with the fracture morphology of untreated Al-Si-Mg alloy shown in Figure 2a [1], dimples of this alloy are deeper and distribute uniformly with a higher number density in different temperature sections, which indicates that the satisfactory ductility can be achieved by the composite modification of Sc and Sr followed by T6 treatment [16]. It is clear that fracture morphology and fracture mode depend on the temperature. Equiaxed dimples uniformly distribute on the fracture of the alloy tested at 20 °C (Figure 2b). With the test temperature increasing (Figure 2c,d), typical microporous aggregate fracture and significant increase of dimples dimension appear, indicating that increasing ductility and plasticity of the matrix [17]. However, gradually apparent tendency of quasi-cleavage step can be observed with the test temperature declining to the low-temperature section (Figure 2e–h), and quasi-cleavage step forms at the temperature of −60 °C (Figure 2h), indicating that the fracture mode converted from ductile to mixed ductile-brittle and quasi-cleavage fracture with the test temperature decreasing [14]. 

**Figure 3:**
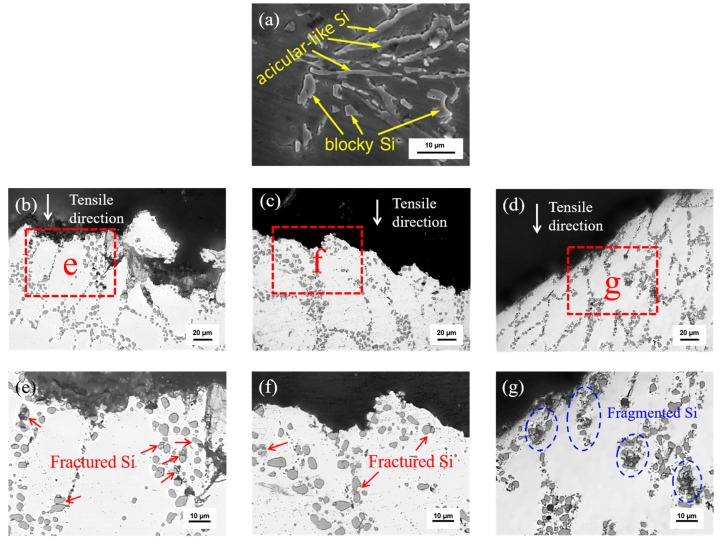
Fracture profiles microstructure of the untreated alloy (**a**) [1] and Sc-Sr composite modified alloy at different temperatures: (**b**) and (**e**) −60 °C, (**c**) and (**f**) 20 °C, (**d**) and (**g**) 200 °C.

Representative fracture profile microstructure of the untreated alloy and Sc-Sr composite modified alloy at different temperatures are shown in Figure 3. From Figure 3a, we could see that acicular-like and blocky eutectic silicon are found in the untreated Al-Si-Mg alloy [1]. However, most of the eutectic silicon after Sc and Sr composite modification are spherical in different temperature sections. Sr element mainly distributes at the growth front of eutectic Si to promote isotropic growth of eutectic Si, and finally converts the morphology of eutectic Si from acicular-like to short fibrous or fine particles [1,2,3]. Meanwhile, crack mainly exists in the eutectic region in low-temperature section but passes through the eutectic region and α-Al matrix in high-temperature section (Figure 3b–d). Besides, fracture profile edges present irregular and sharp shapes in low-temperature section but a relatively flat shape in high-temperature section. At higher magnification (Figure 3e,g), eutectic silicon with larger aspect ratio fractured, and the fracture types are different at different temperatures. Meanwhile, the number of fractured silicon particles at −60 °C is significantly higher than that of 20 °C (Figure 3e,f). Moreover, it can be clearly seen that a large number of eutectic silicon particles fragmented at 200 °C (Figure 3g). According to damage theory, silicon particles will crack when their internal stress approaches the fracture stress [18]. During the plastic deformation procedure, stress concentration is proportional to the aspect ratio of the particles, leading to the fragment of the elongated silicon particles [19]. In the low-temperature section, strength and hardening rate of the alloy increase with the plasticity and ductility decreasing, leading to higher stress state and increased number of fractured silicon particles [20]. In high-temperature section, the matrix is more prone to deform [4], due to the lower elongation, the eutectic Si particles cannot produce such a large deformation as the matrix, leading to a large proportion of silicon particles fragmenting to pieces in the high-temperature section [21]. In addition, in the low-temperature section, many eutectic silicon particles dominate the path of crack propagation; however, in the high-temperature section, fragmented eutectic silicon particles cannot change the propagation path of the cracks, and the cracks can propagate relatively freely, which is the reason for fracture profile micromorphology differences at different temperatures.

### 3.3. Mechanism Analysis

**Figure 4:**
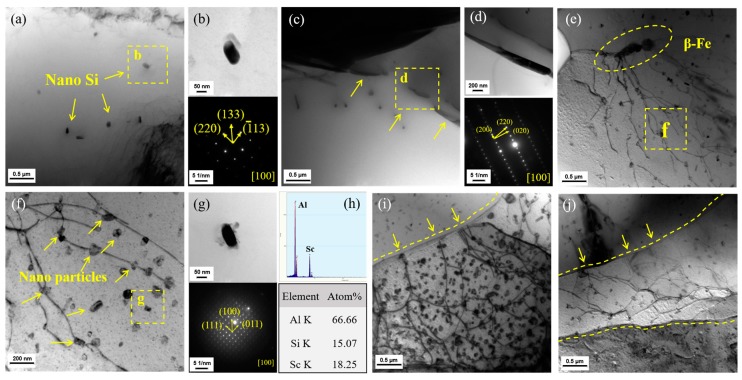
TEM photographs and phase determination of the specimens tested at different temperatures: (**a**,**b**) −60 °C, (**c**,**d**) 20 °C, (**e**–**j**) 200 °C.

Figure 4 shows the representative microstructures of the precipitates (acquired in [100]_Al_ direction) formed at different temperatures. For the specimen tested at −60 °C (Figure 4a), a small number of nanoparticles distributed in the matrix, and the corresponding selected area electron diffraction (SAED) results showed that the nanoparticles were eutectic silicon with a typical ordered (220) (133) (−113) lattice structure (Figure 4b). When the test temperature increased to 20 °C, besides the eutectic silicon, acicular Fe phase was observed in the eutectic area (Figure 4c). SEAD results exhibited a typical ordered structure of β-Al_9_Fe_2_Si_2_ (β-Fe) phase (Figure 4d). It is known that dilute Sr element will cause a coarse flake β-Fe phase to break up and partially dissolve, leading to the formation of acicular β-Fe phase [22]. Moreover, as can be seen from Figure 4e, there is an observative high-density dislocations at the ends of acicular β-Fe phase. Meanwhile, β-Fe phase, as a harmful phase in Al-Si-Mg alloy, can easily cause stress concentration during deformation, which induces the dislocations [23]. Therefore β-Fe phase may serve as the dislocation source when the matrix is subjected to plastic deformation. Under the higher magnification of the specimen tested at 200 °C (Figure 4f), in addition to the aforementioned nano Si particles, a large number of spherical precipitations distribute among the dislocation networks. EDS point scanning and SAED results are shown in Figure 4g,h; the results indicate that the Sc-containing phase with Al: Sc atom ratio close to 3:1 is Al_3_Sc phase. Al_3_Sc phase with L1_2_ structure possesses full coherency with the matrix and lower coarsening rates, which is beneficial to the YS of alloy at high temperature [24,25,26]. Moreover, it can be obviously observed that dislocations pass through the diffusely distributed nano Al_3_Sc particles, meaning typical pining effect, which can hinder the movement of dislocations during the subsequent plastic deformation. Besides the pining effect, it can also be observed that some dislocations end at the grain boundaries (Figure 4i,j). During the plastic deformation under the synergistic influence of temperature and stress, a large number of dislocations originate from the dislocation source multiply and move along the glide plane, but the second phase particles act as obstacles of dislocations, results in tangle and pile-up of dislocation. High-density dislocation network constitutes the deformation substructure, and the interaction between substructure and nanoparticles causes the strengthening of the matrix [27,28]. 

In general, YS is determined by the presence and dimension of obstacles that hinder the motion of dislocations in the matrix [29,30]. Alloy, second phase particles including Al_3_Sc and nano Si particles can lead to Orowan strengthening and thermal expansion strengthening. Orowan strengthening is caused by the resistance of closely spaced hard particles to the passing of dislocations, which is a major factor in aluminum alloys [31]. Meanwhile, when the thermal expansion coefficients of reinforcement particles are significantly different from the matrix, thermal deformation will be produced under the synergistic influence of temperature and stress, and dislocation density will increase, which leads to thermal mismatch strengthening [32]. In addition, the formation of substructure will further hinder the motion of dislocations [33].

Orowan model, thermal mismatch model and Hall-patch model were applied to give a theoretical correlation between the stability of the YS, precipitate phase and deformation substructure in high-temperature section; $\sigma_{s}$ can be expressed as,

(1) $$\sigma_{s}=\sigma_{m}+\Delta \sigma_{orowan}+\Delta \sigma_{TMS}+\Delta \sigma_{b}$$

where the $\sigma_{m}$ is the YS of the aluminum matrix at different temperatures, $\Delta \sigma_{orowan}$ is the strengthening effect caused by Orowan strengthening, $\Delta \sigma_{TMS}$ is the strengthening effect caused by thermal mismatch between the matrix and reinforcement particles, $\Delta \sigma_{b}$ is the strengthening effect caused by substructure.

Orowan strengthening effect can be expressed as [21,27,34],

(2) $$\Delta \sigma_{orowan}=\frac{0.13G_{m}b}{\lambda }ln\frac{d_{p}}{2b}$$

where $G_{m}$ is the shear modulus of the matrix, b is the Burgers vector of the matrix, λ is the interparticle spacing, $d_{p}$ is the nanoparticle size, which can be expressed as,

(3) $$\lambda =d_{p}\left[{\left(\frac{1}{2V_{p}}\right)}^{\frac{1}{3}}-1\right]$$

where $V_{p}$ is the volume fraction of the second phase nanoparticles.

Thermal mismatch strengthening can be expressed as,

(4) $$\Delta \sigma_{TMS}=kG_{m}b\rho_{d}{}^{\frac{1}{2}}$$

where k is constant and equals to 1.25, $\rho_{d}$ is the increased density of dislocation.

Arsenault et al. suggested that $\rho_{d}$ can be expressed as [32,35],

(5) $$\rho_{d}=\frac{BV_{p}\epsilon }{b\left(1-V_{p}\right)}\frac{1}{d_{p}}$$

where B is a geometric constant, ϵ is the mismatch strain due to the different thermal expansion coefficients between the matrix and nanoparticles, which can be expressed as,

(6) ϵ=Δα·ΔT

where Δα is the difference of thermal expansion coefficients between the matrix and nanoparticles, and ΔT is the difference between the room temperature and test temperature.

Modified Hall–Petch model with consideration of second phase pinning can be expressed as follows [36,37,38],

(7) $$\Delta \sigma_{b}=\sigma_{\infty }+kd^{-1/2}=\sigma_{\infty }+KG_{m}\sqrt{b/\left(\frac{4d_{p}}{3V_{p}}\right)}$$

where $\sigma_{\infty }$ is the YS of the single crystal, and typical values for FCC metals are $\sigma_{\infty }\approx 10^{-4}G_{m}$ and $K=\frac{k}{\left(G_{m}\sqrt{b}\right)}≅0.05\sim 0.5\;$.

Substituting Equations (2) to (7) into Equation (1) the following equation about $\sigma_{s}$ can be obtained,

(8) $$\sigma_{s}=\sigma_{m}+\frac{0.13G_{m}b}{d_{p}\left[{\left(\frac{1}{2V_{p}}\right)}^{\frac{1}{3}}-1\right]}ln\frac{d_{p}}{2b}+kG_{m}b{\left[\frac{V_{p}}{\left(1-V_{p}\right)}\frac{B\Delta \alpha \Delta T}{bd_{p}}\right]}^{\frac{1}{2}}+\left[\sigma_{\infty }+KG_{m}\sqrt{b/\left(\frac{4d_{p}}{3V_{p}}\right)}\right]$$

According to the Equation (8), $G_{m}$, b, B, K, k, $\sigma_{\infty }$ are constants that independent of test temperature; hence, it can be deduced that the YS of the alloy is mainly determined by the volume fraction of reinforcement nanoparticles, nanoparticle size, substructure size and the difference of thermal expansion coefficients between the aluminum matrix and nanoparticles. 

In the high-temperature section, the volume fraction $V_{p}$ of second phase particle including Al_3_Sc and nano-silicon and the nanoparticle size $d_{p}$ can be statistically derived from TEM photographs equal to 2.01%–2.04% and 90.46–92.95 nm, respectively, from which it could be seen that $V_{p}$ increase obviously compared to medium-temperature section, and $d_{p}$ of two kinds of particles is typically under 100 nm, and 100 nm is the most desirable particle size for the second phase strengthening [34,39]. Therefore, the Orowan strengthening $\Delta \sigma_{orowan}$ improves significantly at high-temperatures section. On the other hand, the thermal expansion coefficient α of the aluminum matrix is quite different from the particles, where $\alpha_{Al}=2.475\times 10^{-5}\;K^{-1}$, $\alpha_{{\mathrm{Al}}_{3}\mathrm{Sc}}=1.675\times 10^{-5}\;K^{-1}$ and $\alpha_{Si}=3.59\times 10^{-6}\;K^{-1}$, meanwhile the $\alpha_{{\mathrm{Al}}_{3}\mathrm{Sc}}$ is exceedingly stable in a temperature range of 25–900 °C [40], which makes thermal mismatch strengthening $\Delta \sigma_{TMS}$ produce a crucial improvement of YS. Similarly, the substructure strengthening $\Delta \sigma_{b}$ synergistically improved by the higher volume fraction $V_{p}$, smaller nanoparticle size $d_{p}$ and the interaction between substructures and particles. As a summary, the last three terms of Equation (8), i.e., three strengthening effects, are greatly improved in high-temperature section. However due to the soft nature of aluminum matrix in high-temperature section [41], YS will decrease partially; the strengthening of YS mentioned above is offset by softening of aluminum matrix, therefore, YS of the alloy presents completely stability in high-temperature section.

In medium-temperature section, test temperature equals to 20 °C, ΔT=0, and the existence of substructure is not observed under TEM; therefore, thermal mismatch strengthening and substructure strengthening have no contribution on the enhancement of YS. Nano silicon particle size can be statistically derived from the TEM photographs, which remain in the range of 90.78–99.75 nm, indicating that the Orowan strengthening $\Delta \sigma_{orowan}$ is the main strengthening effect in this temperature section. The strengthening effect presents obviously insufficient comparisons with high-temperature section, but the YS of aluminum matrix $\sigma_{m}$ keeps at a high level [42], leading to stable YS compared with the high-temperature section. For the low-temperature section, YS of aluminum matrix $\sigma_{m}$ increased due to the decrease of the temperature [11], and only a small quantity of nano Si particles appears, meaning weak Orowan strengthening effect, leading to the mild increasing of YS with decreasing temperature.

**Figure 5:**
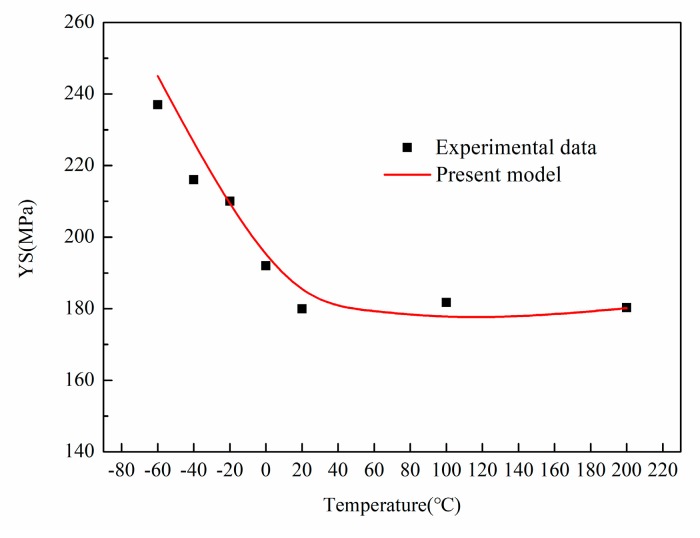
A comparison of the theoretical model and the experimental data at different temperatures.

For the verification of the theoretical model, a quantitative data fitting is performed based on the predicted value and the experimental value. A good agreement between the model prediction and the experimental value could be seen in Figure 5, and the variation in the volume fraction of nanoparticles, generation of substructure and decrease of the YS of aluminum matrix have been taken into consideration.

## 4. Conclusions

Mechanical performances of T6 treated Al-7.12Si-0.36Mg-0.2Sc-0.005Sr alloy at the temperature range of −60 °C to 200 °C were investigated and the following conclusions are obtained. The new designed alloy possesses satisfactory thermal stability; it presents a completely stable yield strength with the elevating of test temperature and a high-level elongation in the whole temperature sections. The fracture mode converts from mixed ductile-brittle fracture and quasi-cleavage fracture in low-temperature section to ductile fracture in high-temperature section. This phenomenon is due to the change of crack propagation and fracture type of eutectic silicon particles. In high-temperature section, β-Fe phase can serve as the dislocation source; Al_3_Sc and nano Si particle have pinning effect to dislocation movement, and dislocations end at the grain boundaries, which results in the formation of deformation substructure, and the interaction between substructure and nanoparticles strengthens the matrix. In high-temperature section, the strengthening effect caused by particles and substructure are offset by the softening of aluminum matrix, therefore, yield strength presents complete stability.
